# Portion size and meal consumption in domesticated dogs: An experimental study

**DOI:** 10.1016/j.physbeh.2019.02.034

**Published:** 2019-05-15

**Authors:** Inge Kersbergen, Alexander J. German, Carri Westgarth, Eric Robinson

**Affiliations:** a Psychological Sciences, University of Liverpool, Liverpool, UK; b School of Health and Related Research, University of Sheffield, Sheffield, UK; c Institute of Veterinary Science, University of Liverpool, Liverpool, UK; d Institute of Ageing and Chronic Disease, University of Liverpool, Liverpool, UK; e Institute of Infection and Global Health, University of Liverpool, Liverpool, UK

**Keywords:** Portion size, Food intake, Feeding behaviour, Dogs, Animals, BCS, Body condition score

## Abstract

Increases in food portion sizes have been identified as a possible contributor to the increased prevalence of obesity in humans. However, little is known about the origin of behavioural tendencies to overeat from larger portion sizes or whether other non-human animals are affected by meal portion size. In the present experimental study, we examined the effect that larger portion sizes have on meal consumption among domesticated dogs (N = 32). Dogs were fed three meals that varied in size on different occasions (150%, 200% and 300% of usual portion size). A repeated measures design was used and food consumption was measured for each meal. Portion size positively affected food consumption, with dogs eating significantly more food as the portion size of meal increased. The effect of portion size on food consumption was also observed when the dogs that finished all available food were excluded from analyses, however not among dogs who did not finish any of the meals. We conclude that the influence larger portions have on food consumption observed in humans is also observed in domesticated dogs. However, it is unclear whether portion size directly biases the amount of food dogs choose to consume, as has been suggested in humans. Further research is now warranted to examine commonalities between human and non-human animal eating behaviour to understand shared behavioural tendencies and their origins.

## 1 Introduction

Human energy intake from food is affected by portion size (i.e., the amount of food that is served on a specific eating occasion): we eat more when offered a larger portion compared to smaller portions. This effect known as the ‘portion size effect’, and has reliably been shown across a range of food types and participant groups [14,35]. Crucially, participants' food intake increases as the portion size increases, even when participants do not finish the entire portion in the smallest portion size condition [29]. Thus, the portion size effect cannot be attributed to people simply running out of food to eat in a meal, rather, food intake appears to be biased by the size of the portion served. Although a well-established phenomenon, the underlying mechanism is unclear. The predominant hypothesis is that portion size communicates information about how much food is socially acceptable or ‘appropriate’ to eat in a particular situation [12,28]. However, given that perceived social appropriateness only partially explains the effect that larger portions have on food intake [17], the exact reasons for the phenomenon remain unclear. Further, although there have been many studies examining whether individual differences moderate the influence of portion size, no sub-groups of participants have been consistently shown to be uninfluenced by portion size when eating [35]. Therefore, it is instead possible that portion size is a core determinant of human energy intake.

Early humans evolved in harsh environments with fluctuating food availability [19]. In such an environment in which food supply is unpredictable and often scarce, maximising energy intake when food is abundant would increase chances of survival [20]. If the portion size effect can be explained by such an evolutionary mechanism, it can be hypothesised that non-human animals who have evolved under similar circumstances will also be influenced by portion size. Domesticated dogs evolved in parallel to humans, with similar genetic traits selected with a positive influence on survival [34]. In the present study, therefore, we moved beyond examining the effect of portion size on human eating behaviour and examined portion size and non-human eating behaviour.

There is a growing interest in studying the eating behaviour of domesticated dogs, because, as with humans, obesity is now highly prevalent among domesticated dogs [10]. Scholars have argued that obesity in pets, such as domesticated dogs, may be driven by the same environmental changes that have caused the human obesity crisis [26,32]. In line with this proposition, dogs are more likely to have obesity if their owners have obesity [24] and if their owners eat a more calorie dense diet [13]. Feeding practises are also thought to be important. For example, dogs fed more frequent meals and snacks are more likely to be overweight [1,3,18]. Further, most people that use a measuring cup to determine the food portion for a meal measure out too much food [8], whilst the use of larger bowls and serving scoops can also increase the likelihood of overfeeding [22]. Despite the work conducted to date, there has been only limited study of a possible influence of meal portion size on canine food intake. In previous work, when forced to make a choice between two bowls, dogs showed a preference for the larger portions compared to smaller portions [21,25]. However, to date, the extent to which domesticated dogs will overeat when served larger portions of food has not been empirically tested and at present we do not know whether, like in humans, portion size biases how much dogs consume during a meal.

Therefore, the current study aimed to investigate the influence that portion size has on food consumption of domesticated dogs. Dogs were fed three meals that varied in portion size (150%, 200% and 300% of usual portion size) in a repeated measures design and food consumption was measured for each meal. We hypothesised that voluntary food intake in dogs would be increased when served larger portions of food.

## 2 Methods

### 2.1 Study design and ethical statement

This was an experimental study with a within-subjects design undertaken in client-owned dogs residing in the North-West of England. The study protocol adhered to the University of Liverpool Animal Ethics Guidelines, and was approved by the University of Liverpool Veterinary Research Ethics Committee (VREC531). Owners of all participating dogs gave written informed consent, and owners received £15 in cash and a £5 shopping voucher as reimbursement for their time.

### 2.2 Recruitment of participants and eligibility criteria

A combination of approaches was used to recruit dog owners in the Liverpool area, including posters in local veterinary practices and dog grooming salons, and advertisements on social media. After registering interest, owners received an information sheet detailing the study procedure and completed a screening questionnaire to assess the dog's eligibility. Dogs were considered eligible for the study if they: 1) lived in a single-dog household, 2) were fed one or two meals a day at approximately the same time of day, 3) had a consistent appetite (owner report), and 4) were at least one year old. Further, dogs were not eligible if they 1) were currently on a weight loss diet, 2) suffered from a medical problem or received medical treatment that might affect appetite, 3) were from a breed known to have a genetic predisposition to excessive food motivation (i.e. Labrador retriever and Flat-coated retriever; [27]), 4) were prone to uncontrolled overeating, or 5) were known to be nervous, fearful, or aggressive around strangers. As this was the first study to investigate the effect of portion size on dogs' food intake, we excluded dogs with a genetic predisposition to excessive food motivation or a history of uncontrolled overeating to reduce the likelihood that all participating dogs would clear their bowl for all portions and to prevent dogs eating to sickness.

### 2.3 Experimental procedures

Three experimental portion size conditions were tested, which were equivalent to 150%, 200%, and 300% of each dogs' typical meal size. The dog's usual food was used in each session, with the order of conditions fully counter-balanced across subjects, with dogs being assigned to one of six possible orders. The in-home study sessions were arranged at the time that each dog was typically fed, with a washout-period of at least 3 days between sessions. To prevent unhealthy weight gain as a result of the study, owners were advised to reduce the dog's normal food intake in the days after the test days, by an amount equivalent to the additional food consumed during the session.

Two researchers attended all study sessions. Written informed consent was obtained from the owner at the start of the first session. During the first session, the researchers asked the owner to measure out the amount of food they would normally give their dog for a meal, to determine the “typical meal portion” of the dog. One researcher then measured out the portion size in accordance with the experimental portion size to be fed (e.g. 150%, 200% and 300%). In most cases, the food was served in the dog's normal feeding bowl. If the normal feeding bowl was not large enough to fit the 300% portion, all portion sizes for that dog were served in a large stainless-steel bowl that the researchers provided. The dog owner placed the food in the normal feeding location, and the dog was then filmed whilst eating. In order not to disrupt the normal feeding behaviour of the dog, the researchers and owner kept as much distance as possible whilst the dog fed and, where possible, left the room. Dogs were deemed to have stopped eating when they left the feeding area or after 20 min had passed since they had last consumed food [33]. The researcher then weighed the amount of food left over.

Owners received a questionnaire regarding their dog's typical eating habits (i.e., food type, meal frequency, and snack frequency) and recent behaviour (Canine Behavioural Assessment and Research Questionnaire (C-BARQ); [15]) to complete once between testing sessions. Once the dog had acclimatised to the researchers, on the final home visit two trained researchers evaluated the dogs' weight status using body condition scoring (BCS). A 9-unit BCS scale was used [9], with dogs classified as underweight (BCS <4/9), ideal weight (BCS 4-5/9), or overweight (BCS >5/9).

### 2.4 Data handling and statistical analysis

All data were recorded in and edited for analyses in SPSS 24 (IBM Corp., 2016). Prior to recruitment, sample size was estimated using a power calculation in G*Power 3.1 [5], based on the ‘medium-sized’ effect of portion size on food intake as seen in humans [14]. This indicated that 28 dogs would be required to detect a medium effect size (f = 0.25) using a repeated-measures ANOVA (α = 0.05, 80% power). We recruited slightly more than this number in order to account for exclusion of test sessions from analyses.

We weighed the food in the feeding bowl and subtracted the weight of the bowl. We calculated how much food dogs consumed by subtracting the weight of any leftover food from the weight of the provided portion and dividing that number by the weight by their typical meal to express the total amount of food eaten in each session as a percentage of their typical meal. This approach was chosen because it accounts for dogs being highly variable in size, consuming different food varieties and thus having different typical meal sizes.

Statistical analyses were performed in SPSS 24.0 [16], with the level of significance set at *p* < .05 for two-sided analyses. To investigate the effect of portion size on food consumption, we conducted multilevel linear regression modelling using maximum likelihood as the estimation method. Portion size was included as a fixed factor predicting the amount eaten as the dependent variable. Sessions were nested within dogs. Significant main effects were followed-up with pairwise comparisons. We conducted two sensitivity analyses to investigate the influence of ‘bowl clearing’ on the results. First, we repeated the main analysis after excluding dogs who finished all of the food provided across conditions (consistent bowl clearers). Then, we included bowl clearing tendencies (i.e. dogs that finished none of the portions vs. dogs that finished at least one portion) and its interaction with portion size as another fixed factor in a multilevel linear regression. To allow for measurement error, a portion was considered to be finished if the dog had left <5% of their typical meal. Finally, we repeated the main analysis with BCS and its interaction with portion size condition as additional predictors to investigate whether the effect of portion size on food intake was moderated by BCS.

Of the sessions recorded, 4 (4%; 2 sessions in the 150%, 1 session in the 200% and 1 session in the 300% portion conditions) were excluded from analysis because the portion size deviated from the required portion by a substantial amount (>20% of typical meal) due to researcher error. A further two sessions (2%; 1 session in the 200% and 1 session in the 300% portion conditions) were excluded because the dog was visibly excited due to the presence of the researcher and ate only a very minimal amount of food (<10% of typical meal). All sessions with valid data were included, as this improves parameter estimation compared to listwise deletion of subjects with any missing data [4]. All dogs had valid data for at least one session. Therefore, the final sample consisted of 90 observations, nested within 32 participants.

Data and analysis protocols are available on https://osf.io/zyejv.

## 3 Results

### 3.1 Study dogs

**Fig. 1:**
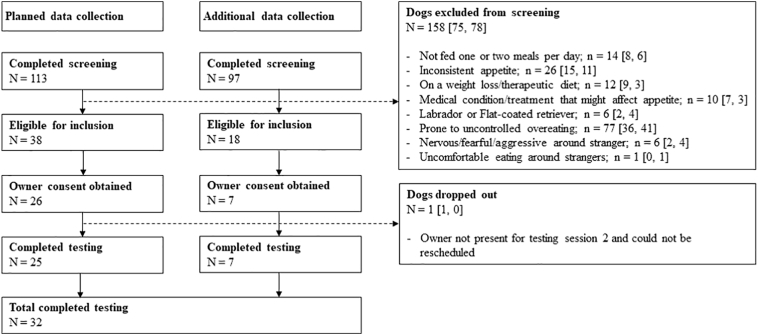
Flow chart outlining eligibility screening and reasons for exclusion. Numbers in brackets indicate *n* for planned data collection and additional data collection, respectively.

A total of 210 dogs were initially assessed for suitability. After initial screening, 56 of these dogs were deemed to be eligible, with follow up to participation and owner consent being obtained for 33 (Fig. 1). Of the participating dogs, 25 completed the initial study by being tested in all 3 conditions. However, preliminary data analysis indicated a larger amount of missing data than intended (see ‘Data exclusion’); we therefore recruited a further 7 dogs before formal data analysis with a total of 32 dogs.

Sixteen dogs were male (9 neutered), and 16 were female (13 neutered), whilst 28 different breeds were represented (list of breeds available in open data on https://osf.io/zyejv). Dogs had a median age of 3 years and 8 months (range: 12 months – 13 years and 3 months) and a median body score condition of 6 (range: 2–9). Based on the BCS assessment, 3 dogs (9%) were classed as underweight, 11 dogs (34%) as ideal weight, and 18 dogs (56%) were classified as overweight. Twenty four dogs (75%) were fed twice daily by their owners and 8 (25%) were fed once daily. Fourteen (44%) dogs were fed dry proprietary food exclusively, 4 dogs (13%) were fed wet proprietary food exclusively, 9 dogs (28%) were fed a combination of wet and dry proprietary food, and 5 dogs (16%) were fed a home-prepared recipe.

### 3.2 Main analysis: effect of portion size condition on consumption

Using multilevel regression modelling, a significant main effect of portion size condition on food consumption was observed (Table 1, Model 1). Pairwise comparisons showed that dogs ate significantly more from the 300% portion size than the 200% portion size, (*p* < .001, d_*z*_ = 0.90) and the 150% portion size (*p* < .001, d_*z*_ = 1.23). Dogs also consumed significantly more from the 200% portion size than the 150% portion size (*p* < .001, d_*z*_ = 0.98). See Table 2 for means and standard deviations.

**Table 1:** Multilevel regression model assessing influence of portion size condition on food intake in dogs, in the full sample (Model 1), after excluding dogs who finished all three portions (Model 2), and in the full sample including bowl clearing as a factor (Model 3).

	Model 1		Model 2		Model 3	
	B (SE)	*p*	B (SE)	*p*	B (SE)	*p*
Fixed components						
Intercept	115 (13.6)		99 (15.6)		146 (8.7)	
150% portion (reference)	–		–		–	
200% portion	34 (10.5)	0.002	29 (12.8)	0.03	47 (10.3)	<0.001
300% portion	88 (10.6)	<0.001	68 (12.8)	<0.001	126 (10.4)	<0.001
Bowl clearing (reference)					–	
Non bowl clearing					−80 (14.2)	<0.001
Non bowl clearing * 200% portion					−35 (17.1)	0.045
Non bowl clearing * 300% portion					−104 (17.2)	<0.001

Random components						
Level 2 variance (Dogs)	4105 (1201.9) [*N* = 32]		3710 (1302.8) [*N* = 23]		425 (212.6) [*N* = 32]	
Level 1 variance (Sessions)	1630 (305.0) [*N* = 90]		1781 (386.4) [*N* = 66]		1009 (188.6) [*N* = 90]

**Table 2:** Food consumption in the different portion size conditions in the full sample and separated by bowl clearing tendencies. Raw means and SDs.

	Full sample (*N* = 32)		Excluding consistent bowl clearers (*N* = 23)		Bowl clearing dogs (*N* = 20)		Non-bowl clearing dogs (*N* = 12)	
	Mean % (SD)	*n*	Mean % (SD)	*n*	Mean % (SD)	*n*	Mean % (SD)	*n*
150% portion	117 (46.2)	30	104 (47.8)	22	145 (22.8)	19	68 (33.7)	11
200% portion	150 (61.6)	30	132 (62.6)	22	192 (14.7)	19	79 (41.4)	11
300% portion	205 (104.7)	30	170 (102.2)	22	272 (44.3)	19	90 (71.6)	11

*Note:* Consumption is expressed as a percentage of participants' typical meal. *n* represents the number of valid sessions in each condition (six sessions were excluded because the portion size deviated from the required portion by a substantial amount due to researcher error or because the dog was visibly excited and ate only a very minimal amount of food).

### 3.3 Sensitivity analysis

#### 3.3.1 Excluding consistent bowl-clearers

After excluding 9 dogs that finished all three portions, a significant main effect of portion size condition on food consumption was still evident (Table 1, Model 2). Using pairwise comparisons, dogs ate more from the 300% portion size than from both the 200% portion size, (*p* = .010, d_*z*_ = 0.63) and the 150% portion size (*p* < .001, d_*z*_ = 0.94). Dogs also consumed more from the 200% portion size than from the 150% portion size (*p* = .003, d_*z*_ = 0.74). See Table 2 for means and standard deviations.

#### 3.3.2 Moderation by bowl clearing tendencies

Twelve dogs were classed as consistent ‘non-bowl clearers’ (finished none of the portions) and 20 dogs finished at least one portion and were therefore classed as a ‘bowl clearer’ in this additional sensitivity analysis. Including bowl clearing as a factor significantly improved model fit compared to the main analysis (χ^2^(3) = 84.67, *p* < .001). There was a significant main effect of portion size condition, a significant main effect of bowl clearing and a significant interaction between bowl clearing and portion size on food consumption (Table 1, Model 3). Overall, non-bowl clearing dogs consumed less food than bowl clearing dogs. Dogs tended to eat more from the 300% portion size than the 200% portion size (bowl clearing dogs *p* < .001, d_*z*_ = 1.92; non-bowl clearing dogs *p* = .60, d_*z*_ = 0.17), and the 150% portion size (bowl clearing dogs *p* < .001, d_*z*_ = 2.30; non-bowl clearing dogs *p* = .18, d_*z*_ = 0.47). Dogs also tended to consume more from the 200% portion size than the 150% portion size (bowl clearing dogs *p* < .001, d_*z*_ = 1.63; non-bowl clearing dogs *p* = .30, d_*z*_ = 0.35). Although these differences were statistically significant among bowl clearing dogs, they were not statistically significant among non-bowl clearing dogs. See Table 2 for means and standard deviations.

#### 3.3.3 Moderation by BCS

Including BCS and its interaction with portion size did not significantly improve model fit compared to the main analysis (χ^2^(3) = 0.72, *p* = .86). BCS was not a direct predictor of food consumption (*F*(1, 30.5) = 0.007, *p* = .93) and did not moderate the effect of portion size on food consumption (*F*(2, 57.35) = 0.36, *p* = .70), indicating no evidence that the effect of portion size on food consumption was associated with dog weight status.

There was no correlation between BCS and the number of portions finished (Spearman's rho = −0.03, *p* = .89). A Chi-Square test with BCS category (underweight; normal weight; overweight) and bowl-clearing status showed no association between weight status and the likelihood of finishing at least one portion during the study (χ^2^(2, *N* = 32) = 0.04, *p* = .98. Among bowl clearers, 2 dogs were classed as underweight (10%), 7 as normal weight (35%) and 11 as overweight (55.0%). Among non-bowl clearers, 1 dog was classed as underweight (8%), 4 as normal weight (33%), and 7 as overweight (58%).

## 4 Discussion

We investigated the effect of portion size on food consumption in domesticated dogs. Our results showed that dogs ate more when they were served larger portions during a single meal. For example, increasing the portion size of meal served from 150% to 300% of the dogs' usual meal size increased food intake during the meal by an average of 88%. These findings indicate that larger portion sizes promote overeating in domesticated dogs. These findings were broadly replicated in the sensitivity analyses when excluding the proportion of dogs that consistently cleared their bowls when eating, but resulted in an attenuation of the influence of portion size on food intake. This suggests that that our main findings are in part explained by a number of dogs readily consuming all food served to them, and it is important to note that this is despite our attempt to exclude known ‘greedy’ dogs through breed genetic tendencies or a history of being prone to uncontrolled over-eating. Further analyses limited to a smaller number of dogs (n = 12) that did not finish any of the meals served to them produced results that were consistent with a linear effect of portion size on food intake (e.g. 22% difference in food intake between smallest and largest portion size condition) but statistically non-significant. Thus, although we can conclude from the present study that domesticated dogs will substantially overeat in response to increased portion size during a single meal, the unexpectedly high frequency of bowl clearing by dogs means that we cannot make convincing conclusions on the extent to which portion size directly biases how much dogs choose to consume during a meal.

Our results are in line with research that shows that humans eat more from larger portions [14,35] and provide the first experimental evidence that the tendency to overeat from larger portions is seen in other non-human animals. These results suggest that both humans and dogs appear to maximise energy intake when food is abundant and this tendency presumably would have been adaptive during much of our evolutionary past [20]. However, it is unclear from the present study whether the effect of portion size on the food intake of dogs reflects a tendency to maximise food intake irrespective of the total amount of food available during a meal (i.e., eating until a physical limit is reached or the portion cleared, whichever comes first), or a tendency to increase food intake when more food is available, even in the absence of bowl-clearing (known as the ‘portion size effect’ as observed in humans). Both of these interpretations would result in dogs being able to consume large amounts of food when readily available and would have likely been adaptive during periods of food scarcity.

### 4.1 Applied relevance

Given that obesity is now worryingly prevalent in domesticated dogs [10], our findings have relevance to owner feeding practices. Our results show that food intake is influenced by the amount of food served and owners have ultimate control over food availability. Given that our results suggest that dogs will substantially overeat from larger portions, it is important that owners weigh out appropriate serving amounts [8] and do not rely on visual judgements alone, as these may result in overfeeding [22]. If the effect of larger portion sizes on food consumption observed during a single meal is maintained long term, as in humans [30,31], variations in owner served portion sizes may be an important factor that explains weight gain in dogs, particularly when taking into account the high number of dogs that are likely to clear their bowl. Given that frequency of feeding is a risk factor for canine obesity [2], we reason that dogs, like humans, are unlikely to fully compensate for increases in energy intake caused by overfeeding. Further research examining the relative contributions of eating frequency, food type and typical portion size on canine obesity may now be informative [1,3].

### 4.2 Limitations and unanswered questions

We used 150%, 200% and 300% of dogs' typical portions, because we reasoned that these would be sufficiently large to prevent dogs from eating all available food, balanced with safety implications of feeding large amounts. This was being applied to a group of dogs who were not reported by their owner to overeat and who were not were from a breed with a genetic predisposition to excessive food motivation [27]. However, despite this, many dogs still cleared their bowls, with the resultant effect that the sensitivity analyses were statistically underpowered when these dogs were excluded. Future research should therefore address this limitation to examine whether portion size biases food consumption in non-human animals.

A second limitation was the fact that we used the dogs' typical portion size (as indicated by owners) to calculate the amount of food served in the different portion sizes, to control for variations in size and breed. Owners may have over or underestimated the dogs' typical portion size, which would lead to the experimental portion sizes being relatively larger or smaller compared to dogs whose owners accurately estimated their dog's typical portion size. Whilst this would not influence within-subjects comparisons, this may confound comparisons between dogs. This approach also did not account for the individual dogs' actual maintenance energy requirement and, even though domestic dogs are reported to be able to consume up to 10% of their own bodyweight of canned food in one sitting [23], it is possible that the larger portions exceeded some dogs' gastric capacity. Future research may benefit from addressing this by using dogs' energy requirements to calculate the baseline portion rather than owners' self-reported typical portion size and taking gastric capacity into account. In addition to variations in typical portion sizes, the brand and type of food used in the study varied widely across individual dogs and some may be more palatable to dogs than others. This study was not powered to detect whether the type of food moderates the effect of portion size on food intake. In humans, the portion size effect does not vary across different food types [35]. Future research examining whether the magnitude of the effect of portion size on dogs' food intake depends on the type of food may be informative.

We only assessed food intake for a single meal. Whilst our results indicate that dogs eat more from larger portions, it is unclear whether they are likely to compensate for the increased energy intake by eating less at a subsequent meal or moving more during the day. We did not formally measure dogs' activity levels, but during visits we anecdotally noticed that most dogs had outside access and were walked regularly. However, considering that owners control the distance and duration of walks, dogs may not be able to compensate much by increasing their energy expenditure. Humans do not fully compensate for greater energy intake from larger portions [31], leading to weight gain [6]. Future research should investigate to what extent dogs compensate for consuming larger portions.

We excluded dogs from breeds with a genetic predisposition to excessive food motivation or a history of uncontrolled overeating, so our findings are limited in generalizability. It would now be valuable to investigate whether reducing dogs' typical portion size is an effective weight management intervention for dogs with excessive food motivation.

Finally, we found no evidence that dog weight status moderated the impact that increased portion size had on feeding behaviour. However, there may be other variables that are associated with the overeating from larger portions. For example, owner feeding practices, such as positive reinforcement for bowl clearing may result in an increased susceptibility to over eat from larger portion sizes in dogs. Comparison with other domesticated animals, such as cats, would be informative, as common owner feeding practices may predict obesity in both dogs and cats [7].

A final consideration is whether our results would differ for related non-domesticated animals (e.g. wolves). Domesticated dogs evolved alongside humans and from an evolutionary perspective it would be informative to know whether any eating tendencies that humans and dogs share are exhibited by similar animals that did not co-evolve with humans. There are a number of animals that appear to be resistant to obesity, even in the face of large food supplies [11], but this is clearly not the case for humans or domesticated dogs. Understanding why may help us to better understand the current human obesity epidemic.

## 5 Conclusions

In summary, our results indicate that larger portions of food increase food intake in domesticated dogs, but further research is required to examine whether, like humans, domesticated dogs' food intake is ‘biased’ by portion size during a meal (i.e., food intake increases when portion size increases, even when comparing portions that are so large that dogs cannot finish either portion).

## Conflicts of interest

ER and IK declare that they have no competing interests. AJG is an employee of the University of Liverpool, but his post is financially supported by Royal Canin. AJG has also received financial remuneration for providing educational material, speaking at conferences, and consultancy work from this company; all such remuneration has been for projects unrelated to the work reported in this manuscript. CW is a consultant for Forthglade Pet Food and has received financial remuneration for work unrelated to this manuscript. CW has also previously received grant funding from WALTHAM/Mars Petcare, for work unrelated to this manuscript. ER has previously received funding from Unilever and the American Beverage Association for unrelated research.

## Funding

The study received internal funding from the School of Psychology at the University of Liverpool. There was no external funding for this study. ER's salary is supported by the Medical Research Council [MR/N000218/1].

## References

[1] Bland I.M., Guthrie-Jones A., Taylor R.D., Hill J. (2009). Dog obesity: owner attitudes and behaviour. Prevent. Vet. Med..

[2] Bland I.M., Guthrie-Jones A., Taylor R.D., Hill J. (2010). Dog obesity: veterinary practices' and owners' opinions on cause and management. Prevent. Vet. Med..

[3] Courcier E.A., Thomson R.M., Mellor D.J., Yam P.S. (2010). An epidemiological study of environmental factors associated with canine obesity. J. Small Anim. Pract..

[4] Dong Y., Peng C.Y.J. (2013). Principled missing data methods for researchers. SpringerPlus.

[5] Faul F., Erdfelder E., Lang A.-G., Buchner A. (2007). G*power: a flexible statistical power analysis program for the social, behavioral, and biomedical sciences. Behav. Res. Methods.

[6] French S.A., Mitchell N.R., Wolfson J., Harnack L.J., Jeffery R.W., Gerlach A.F., Pentel P.R. (2014). Portion size effects on weight gain in a free living setting. Obesity.

[7] German A.J. (2015). Style over substance: what can parenting styles tell us about ownership styles and obesity in companion animals?. Br. J. Nutr..

[8] German A.J., Holden S.L., Mason S.L., Bryner C., Bouldoires C., Morris P.J., Biourge V. (2011). Imprecision when using measuring cups to weigh out extruded dry kibbled food. J. Anim. Physiol. Anim. Nutr..

[9] Laflamme D. (1997). Development and validation of a body condition score system for dogs. Canine Pract..

[10] German A.J., Woods G.R.T., Holden S.L., Brennan L., Burke C. (2018). Dangerous trends in pet obesity. Vet. Rec..

[11] Halsey L.G. (2018). Keeping slim when food is abundant: what energy mechanisms could be at play?. Trends Ecol. Evol..

[12] Haynes A., Hardman C.A., Makin A.D.J., Halford J.C.G., Jebb S.A., Robinson E. (2019). Visual perceptions of portion size normality and intended food consumption: a norm range model. Food Qual. Prefer..

[13] Heuberger R., Wakshlag J. (2011). The relationship of feeding patterns and obesity in dogs. J. Anim. Physiol. Anim. Nutr..

[14] Hollands G.J., Shemilt I., Marteau T.M., Jebb S.A., Lewis H.B., Wei Y., Ogilvie D. (2015). Portion, package or tableware size for changing selection and consumption of food, alcohol and tobacco. Cochrane Database Syst. Rev..

[15] Hsu Y., Serpell J.A. (2003). Development and validation of a questionnaire for measuring behavior and temperament traits in pet dogs. J. Am. Vet. Med. Assoc..

[16] Corp I.B.M. (2016). IBM SPSS Statistics for Windows, Version 24.0.

[17] Kerameas K., Vartanian L.R., Herman C.P., Polivy J. (2015). The effect of portion dize and unit size on food intake: unit bias or segmentation effect?. Health Psych..

[18] Kienzle E., Bergler R., Mandernach A. (1998). A comparison of the feeding behavior and the human-animal relationship in owners of normal and obese dogs. J. Nutr..

[19] King B.M. (2013). The modern obesity epidemic, ancestral hunter-gatherers, and the sensory/reward control of food intake. Am. Psychol..

[20] Lieberman L.S. (2006). Evolutionary and anthropological perspectives on optimal foraging in obesogenic environments. Appetite.

[21] Marshall-Pescini S., Prato-Previde E., Valsecchi P. (2011). Are dogs (Canis familiaris) misled more by their owners than by strangers in a food choice task?. Anim. Cogn..

[22] Murphy M., Lusby A.L., Bartges J.W., Kirk C.A. (2012). Size of food bowl and scoop affects amount of food owners feed their dogs. J. Anim. Physiol. Anim. Nutr..

[23] National Research Council (2006). Feeding behavior in dogs and cats. Nutrient Requirements of Dogs and Cats.

[24] Nijland M.L., Stam F., Seidell J.C. (2010). Overweight in dogs, but not in cats, is related to overweight in their owners. Public Health Nutr..

[25] Prato-Previde E., Marshall-Pescini S., Valsecchi P. (2008). Is your choice my choice? The owners' effect on pet dogs' (Canis lupus familiaris) performance in a food choice task. Anim. Cogn..

[26] Pretlow R.A., Corbee R.J. (2016). Similarities between obesity in pets and children: the addiction model. Br. J. Nutr..

[27] Raffan E., Dennis R.J., O'Donovan C.J., Becker J.M., Scott R.A., Smith S.P., O'Rahilly S. (2016). A deletion in the canine POMC gene is associated with weight and appetite in obesity-prone Labrador retriever dogs. Cell Metab..

[28] Robinson E., Kersbergen I. (2018). Portion size and later food intake: evidence on the “normalizing” effect of reducing food portion sizes. Am. J. Clin. Nutr..

[29] Rolls B.J., Morris E.L., Roe L.S. (2002). Portion size of food affects energy intake in normal-weight and overweight men and women. Am. J. Clin. Nutr..

[30] Rolls B.J., Roe L.S., Meengs J.S. (2006). Larger portion sizes lead to a sustained increase in energy intake over 2 days. J. Am. Diet. Assoc..

[31] Rolls B.J., Roe L.S., Meengs J.S. (2007). The effect of large portion sizes on energy intake is sustained for 11 days. Obesity.

[32] Sandøe P., Palmer C., Corr S., Astrup A., Reinhard Bjørnvad C. (2014). Canine and feline obesity: a one health perspective. Vet. Rec..

[33] Smith J.C., Rashotte M.E., Austin T., Griffin R.W. (1984). Fine-grained measures of dogs' eating behavior in single-pan and two-pan tests. Neurosci. Biobehav. Rev..

[34] Wang G.D., Zhai W., Yang H.C., Fan R.X., Cao X., Zhong L., Zhang Y.P. (2013). The genomics of selection in dogs and the parallel evolution between dogs and humans. Nat. Commun..

[35] Zlatevska N., Dubelaar C., Holden S.S. (2014). Sizing up the effect of portion size on consumption: a meta-analytic review. J. Mark..

